# Analyzing dissemination, quality, and reliability of Chinese brain tumor-related short videos on TikTok and Bilibili: a cross-sectional study

**DOI:** 10.3389/fneur.2024.1404038

**Published:** 2024-10-18

**Authors:** Ren Zhang, Zhiwei Zhang, Hui Jie, Yi Guo, Yi Liu, Yuan Yang, Chuan Li, Chenglin Guo

**Affiliations:** ^1^Department of Thoracic Surgery and Institute of Thoracic Oncology, West China Hospital, Sichuan University, Chengdu, China; ^2^West China School of Medicine, Sichuan University, Chengdu, China; ^3^Department of Neurosurgery, West China Hospital, Sichuan University, Chengdu, China

**Keywords:** brain tumors, short videos, XGBoost, DISCERN, Global Quality Score, social media

## Abstract

**Background:**

As the Internet becomes an increasingly vital source of medical information, the quality and reliability of brain tumor-related short videos on platforms such as TikTok and Bilibili have not been adequately evaluated. Therefore, this study aims to assess these aspects and explore the factors influencing the dissemination of such videos.

**Methods:**

A cross-sectional analysis was conducted on the top 100 brain tumor-related short videos from TikTok and Bilibili. The videos were evaluated using the Global Quality Score and the DISCERN reliability instrument. An eXtreme Gradient Boosting algorithm was utilized to predict dissemination outcomes. The videos were also categorized by content type and uploader.

**Results:**

TikTok videos scored relatively higher on both the Global Quality Score (median 2, interquartile range [2, 3] on TikTok vs. median 2, interquartile range [1, 2] on Bilibili, *p* = 1.51E-04) and the DISCERN reliability instrument (median 15, interquartile range [13, 18.25] on TikTok vs. 13.5, interquartile range [11, 16] on Bilibili, *p* = 1.66E-04). Subgroup analysis revealed that videos uploaded by professional individuals and institutions had higher quality and reliability compared to those uploaded by non-professional entities. Videos focusing on disease knowledge exhibited the highest quality and reliability compared to other content types. The number of followers emerged as the most important variable in our dissemination prediction model.

**Conclusion:**

The overall quality and reliability of brain tumor-related short videos on TikTok and Bilibili were unsatisfactory and did not significantly influence video dissemination. Future research should expand the scope to better understand the factors driving the dissemination of medical-themed videos.

## 1 Introduction

Brain tumors consist of a group of intracranial neoplastic lesions originating from various cell types. In China, the mortality rate of central nervous system (CNS) tumors in 2020 was 3.86 per 100,000 population, with a total of 1710.90 years of life lost, causing a huge burden on patients and society (1). The clinical manifestations of brain tumors depend on their size, location, and biological behavior and can include deficits in the affected brain area or cranial nerve, cognitive impairment, seizures, and increased intracranial pressure, which may worsen as the tumor grows. Magnetic resonance imaging (MRI) is typically used when a brain tumor is suspected (2). Given the diversity of brain tumors, treatment strategies range from observation, pharmacotherapy, and radiotherapy to stereotactic biopsy and surgical resection, depending on the patient’s overall health, preoperative diagnosis, and potential postoperative pathology (3). Therefore, timely detection, accurate diagnosis, and appropriate management are critical for neurosurgeons to maximize patient outcomes in brain tumor cases.

In addition to medical journals and consultations with doctors, the Internet has become an important source of medical information for the public due to its rapid expansion. The types of media are continuously evolving with advances in information dissemination and storage technology (4). Concerns have been raised about the quality and reliability of online medical information. Patients are at risk of making decisions about their health issues based on low-quality and inaccurate sources of information (5). Addressing incorrect or inappropriate online medical information would cost professional health providers more time and effort (6). Systematic investigation and analysis of online medical information are fundamental to help understand the current situation and further set standards and protocols to improve the quality of online medical information. Several tools have been developed to evaluate and grade the medical content. For example, Silberg et al. (7) proposed a basic standard for the quality of Internet-based sources of medical information. The DISCERN instrument was developed to judge the quality of written information on medical treatment (8). The Quality Evaluation Instrument and Global Quality Score (GQS) have been used to evaluate the information sources of inflammatory bowel disease, and poor quality of websites has been reported (9).

Nevertheless, a large amount of low-quality Internet-based medical information could mislead the public (10, 11). One study of trends in brain tumors on social media (Facebook, YouTube, Twitter, and Instagram) suggested a robust patient community online (12). Further research on the quality of medical information is warranted.

Nowadays, short videos have become popular worldwide. Compared with traditional online videos, short videos are less time-consuming and easier to access and interact with while providing attractive audiovisual content compared to tweets and blogs. The morbidity of CNS tumors ranks 15th among all cancers in China, which receives relatively less attention than other neoplastic disorders affecting a larger population of patients (13). The quality of online videos about meningioma has been investigated and found to be poor (14). Additionally, the same issue of quality and reliability for short videos should not be overlooked. As a new type of social media, short videos are usually made and uploaded by users spontaneously, lacking professional reviews for quality and reliability. Moreover, the recommendation system based on personal preferences makes it easier for users to obtain biased information (15).

Machine learning techniques, such as eXtreme Gradient Boosting (XGBoost), have been widely applied across multiple fields. XGBoost is a powerful and efficient machine learning algorithm known for its exceptional performance in regression, classification, and ranking tasks. It operates by creating an ensemble of decision trees, where each tree corrects the errors of the preceding ones, a process known as boosting. This iterative improvement allows XGBoost to effectively model complex, non-linear relationships between features, making it particularly suited for datasets with intricate interactions (16).

In the medical domain, XGBoost has been successfully applied to numerous challenges, such as the early diagnosis of COVID-19 from X-ray images, predicting restenosis following percutaneous coronary intervention, predicting metabolic syndrome, and classifying diabetes. These applications demonstrate its versatility and robustness in handling medical data (17–20). The success of XGBoost in these areas highlights its potential for analyzing and interpreting the dissemination, quality, and reliability of medical-themed short videos, offering a novel and powerful approach compared to traditional models.

While XGBoost excels in handling complex datasets, understanding the predictions of such models is essential, especially in the medical field, where the interpretability of model outputs can directly influence clinical decision-making. SHapley Additive explanation (SHAP) provides a consistent and theoretically sound method for explaining individual predictions (21). SHAP works by evaluating all possible feature combinations and calculating each feature’s marginal contribution across these combinations, resulting in a comprehensive measure of feature importance (21). SHAP also helps explain how changes in input variables affect outcomes for each sample (22). SHAP has been extensively utilized for interpreting complex machine learning models, such as in forecasting bitcoin and predicting toluene behavior in the atmosphere (23, 24).

Many studies have focused on the quality of videos on different diseases on traditional video platforms such as YouTube (25, 26). However, despite the rise in health information dissemination through short video platforms over the past decade, video content on these platforms has not been sufficiently investigated (27, 28). Based on platforms such as TikTok, previous research has extensively analyzed the quality of medical information in short videos across various health topics, including liver cancers (29), gastric cancers (30), *Helicobacter pylori* infection (31), gallstones (32), non-alcoholic fatty liver disease (33), dementia (34), monkeypox (35), and diabetes (36). However, no studies have specifically analyzed short videos related to brain tumors in China. In addition, previous studies mainly focused on the quality and reliability of videos featuring medical information while neglecting the dissemination effect of videos (29, 37–39). Although some studies have explored video propagation, they primarily employed basic statistical methods, such as multiple linear regression (40, 41). Notably, there is a significant research gap in understanding the dissemination dynamics of brain tumor-related videos, particularly how these videos spread on social media platforms and what factors contribute to their reach and engagement. Addressing this gap is essential for developing more effective strategies to improve the impact of medical-themed short videos.

This study aims to comprehensively evaluate the quality, reliability, and dissemination of brain tumor-related short videos on TikTok and Bilibili. Subgroup analysis is intended to explore diverse characteristics influencing video quality and reliability; comparative analysis between TikTok and Bilibili aims to uncover platform-specific trends. By employing machine learning techniques, our study offers a novel approach to identifying and interpreting factors influencing the dissemination of brain tumor-related videos, informing the production of such videos.

## 2 Methods

### 2.1 Video retrieval

This cross-sectional study chose TikTok and Bilibili on the Chinese Internet as the video sources. TikTok and Bilibili are among China’s most representative video platforms (37). TikTok has over 100 million users worldwide across over 150 countries. It is known for rapid content dissemination and high user engagement through short videos. Bilibili is a Chinese video platform that integrates entertainment and education, providing millions of active users per month, including patients and doctors, with diverse content and deep community interaction to learn health-related knowledge (29, 37, 42). Through videos on TikTok and Bilibili, users can access a wide range of health-related videos, including brain tumor-related videos. To obtain brain tumor-related short videos, the keyword “脑肿瘤” (brain tumor in Chinese) was entered into the search box of the web versions of TikTok (43) and Bilibili (44) separately. The same search strategy was applied for each video source. Videos were ranked based on comprehensive sorting to stimulate the search results by the public (29). The default settings of the website were maintained, and the anonymous status was preserved to minimize potential bias due to the personal preference of the researcher. Videos longer than 10 min, videos without Chinese subtitles or narrations, videos with content identical to those already selected, videos without medical information, and completely unrelated videos such as web advertising were ruled out. The search was conducted between 17th Dec 2023 and 19th Dec 2023, and the top 100 videos from each platform were selected as the same sample size adopted by many other similar studies (29, 45).

Basic information was documented, including statistics that characterized video dissemination. For each video, the title, upload date, length, and the number of likes, comments, shares, and saves were recorded, and the number of days since uploading was calculated. For content in the video, the presence of people and the addition of background music (BGM) and subtitles were noted. For uploaders, their names, identity authentication status, number of followers, and number of uploaded videos were recorded.

### 2.2 Video classification

Labels were assigned to videos based on their content and the characteristics of uploaders to further classify the videos. The videos were categorized as (1) basic knowledge of the disease, (2) surgical demonstrations, (3) medical research or professional education, (4) case reports and related discussions, (5) clips of news or documentary, and (6) others. The same categorization of uploaders was adopted by Zheng et al. (29). Specifically, the uploaders were categorized based on (1) whether they are specialized in medicine and (2) whether they are individuals or institutions. Professional individuals were further classified as (1) brain tumor-related modern medicine specialists, (2) other modern medicine specialists, (3) traditional medicine specialists, and (4) other medical specialists. Supplementary Table S1 describes the names of subgroups and their criteria.

### 2.3 Quantitative evaluation of video quality and reliability

The GQS and the first part of the DISCERN instrument (the DISCERN reliability instrument) were employed to assess the quality and reliability of each short video, respectively. The GQS is a widely recognized and validated instrument for accessing quality videos featuring medical information (29, 32, 38, 46). The GQS evaluates the quality of videos according to comprehensiveness, usability, and overall quality (9). The GQS is a subjective 5-point scale that evaluates online medical information based on quality, flow, integrity of information, and ease of use (9). The DISCERN is a widely validated and applied instrument for evaluating the reliability of health-related content in videos (8, 38, 46). The DISCERN provides a method for rating the quality of the obtained medical information with fifteen key questions and an overall quality rating question; these questions focus on the reliability, treatment options, and overall quality of the medical information (8). Each question is rated from one (definite no) to five (definite yes) to assess the reliability of publications (8). The complete descriptions of GQS and the DISCERN reliability instrument were presented in Supplementary Tables S2, S3.

To ensure consistency in evaluation, the videos were collected by Ren Zhang and randomly sorted by Yi Liu before two experienced surgeons, Yuan Yang, and Chenglin Guo, independently assessed the quality and reliability of these videos. All researchers then reviewed the results.

### 2.4 Identifying dissemination metrics with XGBoost

We extracted dissemination metrics for each video, including the number of likes, comments, shares, and saves. Likes indicate immediate positive reactions; comments reflect deeper user interaction and feedback; shares represent users’ endorsement and willingness to further disseminate the content (41). These metrics represent social media engagement rates, reflecting how well-shared content resonates with the audience’s preferences, relevance, and interests (40). Social media industry standards consider these metrics to measure user engagement with social media content (47). To better understand the dissemination effect of short videos, the XGBoost algorithm was used to investigate the potential factors associated with the number of likes on brain tumor-related short videos, as it has shown consistent and positive associations with various types of user engagement (48). To optimize the performance of our XGBoost model and mitigate overfitting, we employed grid search for hyperparameter tuning (49). The key hyperparameters selected for tuning included the learning rate and maximum depth (16, 50). For each session of hyperparameter tuning, 3-fold cross-validation was implemented, with classification error serving as the performance metric (49). This approach ensured that the selected hyperparameters were robust across different subsets of the data. A combination of hyperparameters with the lowest classification error was adopted to analyze dissemination factors further.

Variables included in this analysis were the year of uploading, days since uploading, video length, type of video content, uploader characteristics (such as the number of followers, the number of uploaded videos, and authentication status), presence of people, use of BGM and subtitles, and two assessment scores. The importance of features in the machine learning model was calculated; the SHAP was employed to explore the contribution of each factor and explain how these factors influence video dissemination (22).

### 2.5 Statistical analysis

All statistical analyses were conducted using R version 4.2.3 (51). Due to their nonnormal distribution, continuous variables were described using the median and interquartile range (IQR). The Wilcoxon rank-sum test was used to compare differences between continuous variables, while the chi-square test was used for discrete variables, with Fisher’s exact test or Yates’ correction applied when appropriate (52).

The kappa consistency test was used to examine the consistency between quality and reliability rating, with a *p*-value of >0.8 indicating acceptable consistency. The R packages “xgboost” (53) and “fastshap” (54) were used to build the dissemination effect model, while “ggplot2” (55) was used to visualize the results. Unless otherwise specified, a two-sided *p*-value of 0.05 was considered the threshold for statistical significance. In the figures, a single * denotes a *p*-value of <0.05, a double * indicates a *p*-value of <0.01, and a triple * signifies a *p*-value of <0.001.

## 3 Results

### 3.1 Basic characteristics of videos

**Figure 1:**
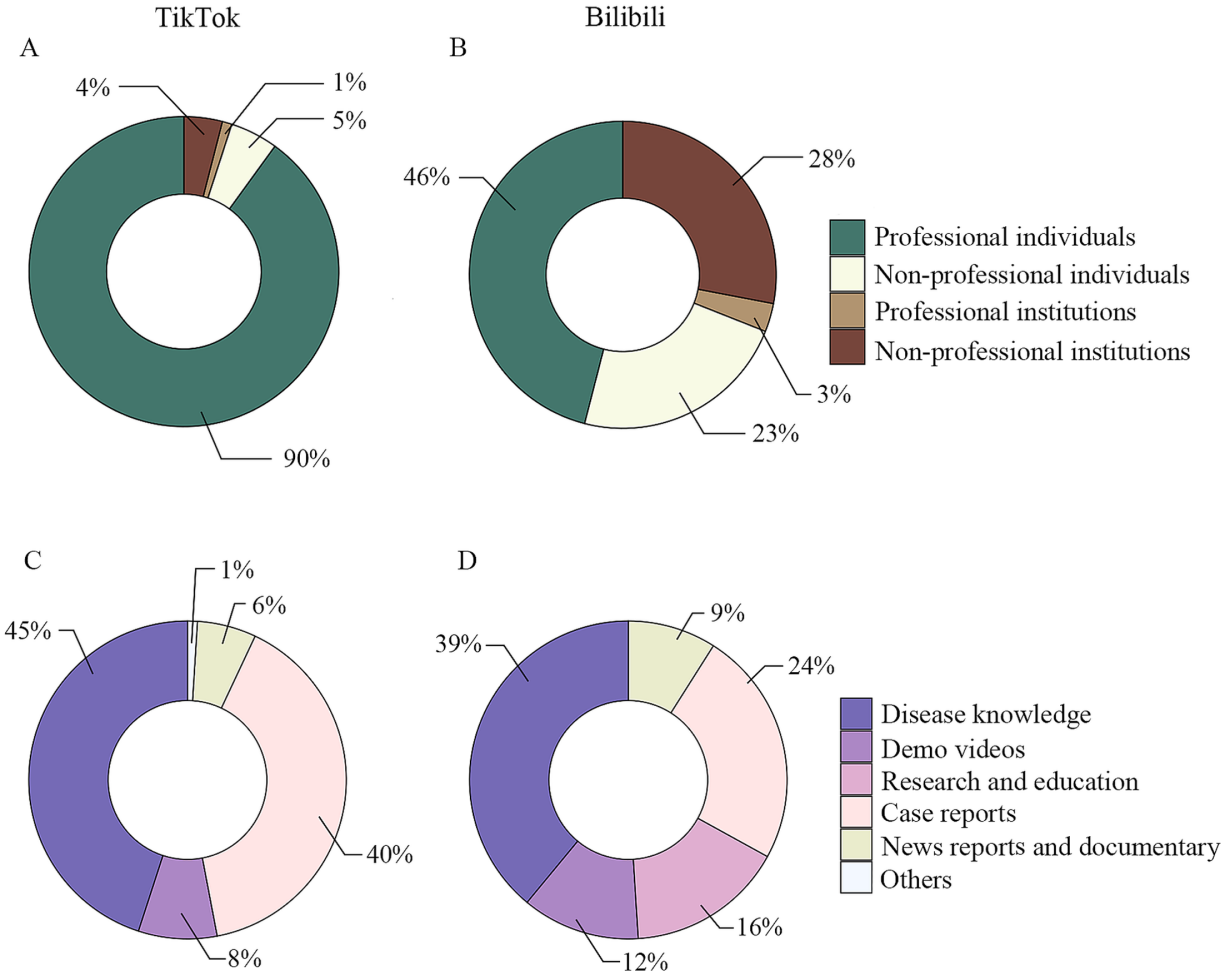
The composition of the classification of uploaders and video content on two platforms. (A) Types of uploaders for short videos on TikTok; (B) types of uploaders for short videos on Bilibili; (C) classification of video content in short videos on TikTok; (D) classification of video content in short videos on Bilibili.

During the selection, 21 videos (19 unrelated videos and two duplicated videos) were excluded on TikTok, while 63 videos (44 unrelated videos, 11 duplicated videos, 5 non-Chinese videos, and 3 videos with unknown uploaders) were excluded on Bilibili. The basic information of the 200 included short videos is listed in Table 1. Regarding the dissemination effect, the number of likes (*p* = 4.72E-21), comments (*p* = 4.41E-23), shares (*p* = 5.04E-20), and saves (*p* = 2.02E-16) were higher on TikTok. Bilibili had longer video durations (*p* = 1.09E-03) and more days since uploading (*p* = 1.06E-02). Regarding video content, the presence of people (*p* = 2.53E-04) and the use of subtitles (*p* = 1.56E-03) were more frequent on TikTok. As for uploader statistics, TikTok had significantly more followers (*p* = 1.21E-13) and authenticated accounts (*p* = 7.11E-31) compared to Bilibili. The composition of the type of uploaders (*p* = 2.49E-15) and content (*p* = 4.25E-05) were significantly different based on our categorization. Most videos were uploaded by individuals. In summary, the majority of the short videos on TikTok were uploaded by professionals (*n* = 90, 90%), while half of the short videos on Bilibili were from uploaders without a medical background (*n* = 51, 51%). Regarding video content, TikTok predominantly featured clinical disease knowledge (*n* = 45, 45%) and case reports (*n* = 40, 40%), with a less diverse variety of content types, as shown in Figure 1. No significant differences were found in the total number of uploaded videos per uploader (*p* = 0.51) and the use of BGM (*p* = 0.56) between the two platforms (Table 1). Together, these data suggested that the content of brain tumor-related short videos varies significantly between the two platforms.

**Table 1:** Baseline of brain tumor-related short videos.

Characteristics	TikTok (*n* = 100)	Bilibili (*n* = 100)	*p* value
Likes, median (IQR)	362 (172, 1247.8)	4 (1, 37.75)	4.72E-21
Saves, median (IQR)	57.5 (24.75, 166)	2 (0, 18.25)	2.02E-16
Comments, median (IQR)	55.5 (17.75, 202.25)	0 (0, 4)	4.41E-23
Shares, median (IQR)	48.5 (13.5, 173.75)	1 (0, 6.25)	5.04E-20
Days since uploading, median (IQR)	285.5 (115, 599.5)	467 (146, 722)	1.06E-02
Length, median (IQR)	67.5 (45, 109)	90 (62.5, 213)	1.09E-03
Followers, median (IQR)	2.6e+04 (7916.8, 1.062e+05)	677.5 (113.5, 1.225e+04)	1.21E-13
Total video count, median (IQR)	284 (114.75, 596.75)	277 (42, 700.75)	0.51
Type of videos, median (IQR)			4.25E-05
1	45 (45%)	39 (39%)	
2	8 (8%)	12 (12%)	
3	0 (0%)	16 (16%)	
4	40 (40%)	24 (24%)	
5	6 (6%)	9 (9%)	
6	1 (1%)	0 (0%)	
Type of uploaders, *n* (%)			1.02E-09
1	90 (90%)	46 (46%)	
2	5 (5%)	23 (23%)	
3	1 (1%)	3 (3%)	
4*	0 (0%)	0 (0%)	
5	4 (4%)	28 (28%)	
Types of professionals, *n* (%)			2.49E-15
1	88 (88%)	30 (30%)	
2	1 (1%)	1 (1%)	
3	1 (1%)	16 (16%)	
4	10 (10%)	53 (53%)	
Authentication, *n* (%)			7.11E-31
0	3 (3%)	84 (84%)	
1	97 (97%)	16 (16%)	
The presence of people, *n* (%)			2.53E-04
0	13 (13%)	36 (35%)	
1	87 (87%)	64 (65%)	
BGM, *n* (%)			0.56
0	39 (39%)	35 (35%)	
1	61 (61%)	65 (65%)	
Subtitles, *n* (%)			1.56E-03
0	4 (4%)	18 (18%)	
1	96 (96%)	82 (82%)	
DISCERN score, median (IQR)	15 (13, 18.25)	13.5 (11, 16)	1.66E-04
GQS score, median (IQR)	2 (2, 3)	2 (1, 2)	1.51E-04

^*^No videos were uploaded by other medical practitioners, so they were removed excluded from further analysis.

IQR, interquartile range; BGM, background music; GQS, Global Quality Score.

### 3.2 Quality and reliability assessments

**Figure 2:**
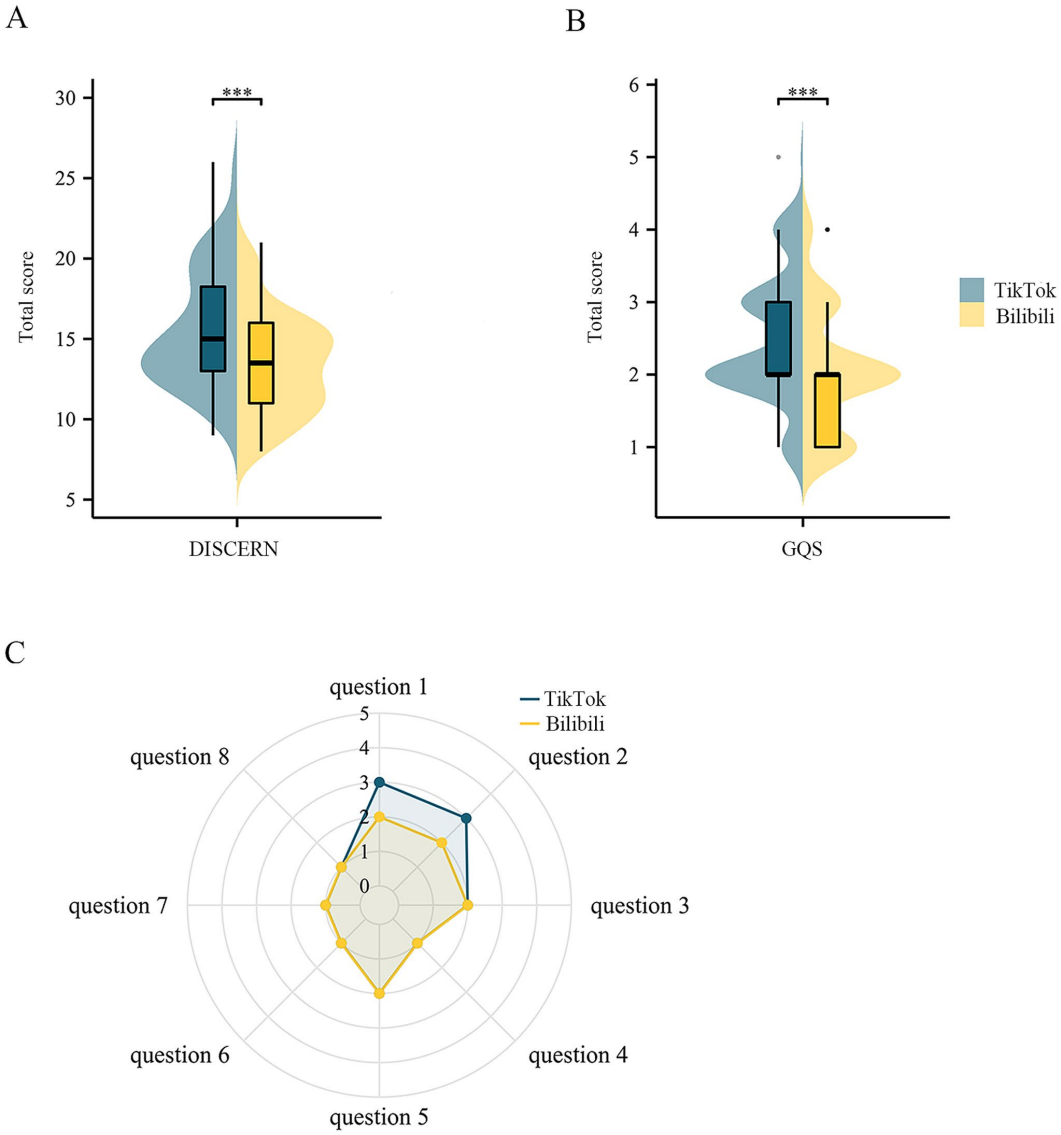
Quality and reliability scores of brain tumor-related short videos. (A) Scores of the DISCERN reliability instrument; (B) scores of the GQS; (C) scores of each question of the DISCERN reliability instrument. GQS, Global Quality Score.

No discrepancies were found by the kappa consistency test for all ratings (*p* > 0.8, Supplementary Table S4), allowing for subsequent data analysis. As presented in Table 1 and Figures 2A,B, the median overall GQS score (2, IQR [2, 3] on TikTok; 2, IQR [1, 2] on Bilibili) and DISCERN reliability score (15, IQR [13, 18.25] on TikTok; 13.5, IQR [11, 16] on Bilibili) of brain tumor-related short videos were below average, although relatively higher quality (*p* = 1.51E-04) and reliability (*p* = 1.66E-04) were found on TikTok. The detailed results of DISCERN reliability are presented in Figure 2C. However, some short videos managed to set a clear aim and achieve it with relevant medical content, and this proportion was higher on TikTok (*p* = 2.20E-02 for question 1; *p* = 1.54E-05 for question 2; *p* = 9.33E-07 for question 3).

**Figure 3:**
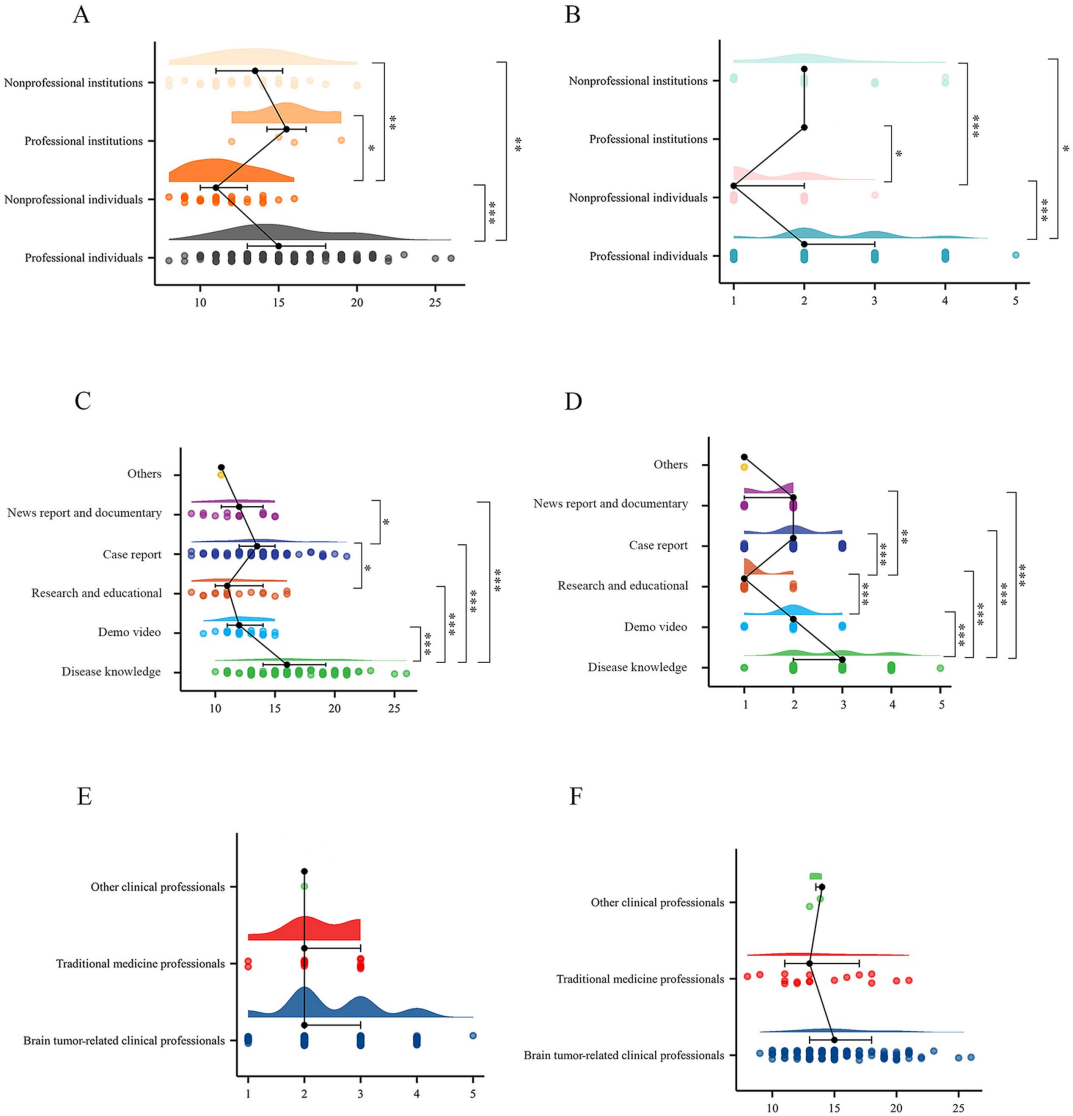
Quality and reliability scores of brain tumor-related short videos in different subgroups. (A) Scores of DISCERN reliability instrument in different types of uploaders; (B) scores of GQS in different types of uploaders; (C) scores of DISCERN reliability instrument in different types of video content; (D) scores of GQS in different types of video content; (E) scores of DISCERN reliability instrument in different types of professional individual uploaders; (F) scores of GQS in different types of professional individual uploaders. GQS, Global Quality Score.

To better explore the quality and reliability of brain tumor-related short videos across different uploader and content categories, videos were further categorized, and subgroup analyses were conducted. The baselines of different subgroups were provided in Supplementary Tables S5, S6. In Figures 3A,B, professional individuals achieved higher DISCERN reliability and GQS scores than nonprofessional individuals (*p* = 4.92E-08 for DISCERN score; *p* = 9.73E-09 for GQS score) and institutions (*p* = 9.08E-03 for DISCERN score; *p* = 2.73E-02 for GQS score). The same phenomenon was observed for professional institutions, although this finding should be interpreted cautiously given the small number of videos uploaded by them. Regarding video content types in Figures 3C,D, disease knowledge introductions had the highest quality and reliability scores, followed by case reports. Among the professional individuals, no videos were uploaded by nurses, technicians, or researchers; thus, the other three types were compared. No significant differences in video quality and reliability were found among the remaining categories, as shown in Figures 3E,F. Overall, although the quality of brain tumor-related short videos might not fully meet expectations, videos focusing on disease knowledge and case reports and those uploaded by professionals demonstrated relatively higher performance.

### 3.3 The dissemination effect of short videos

**Figure 4:**
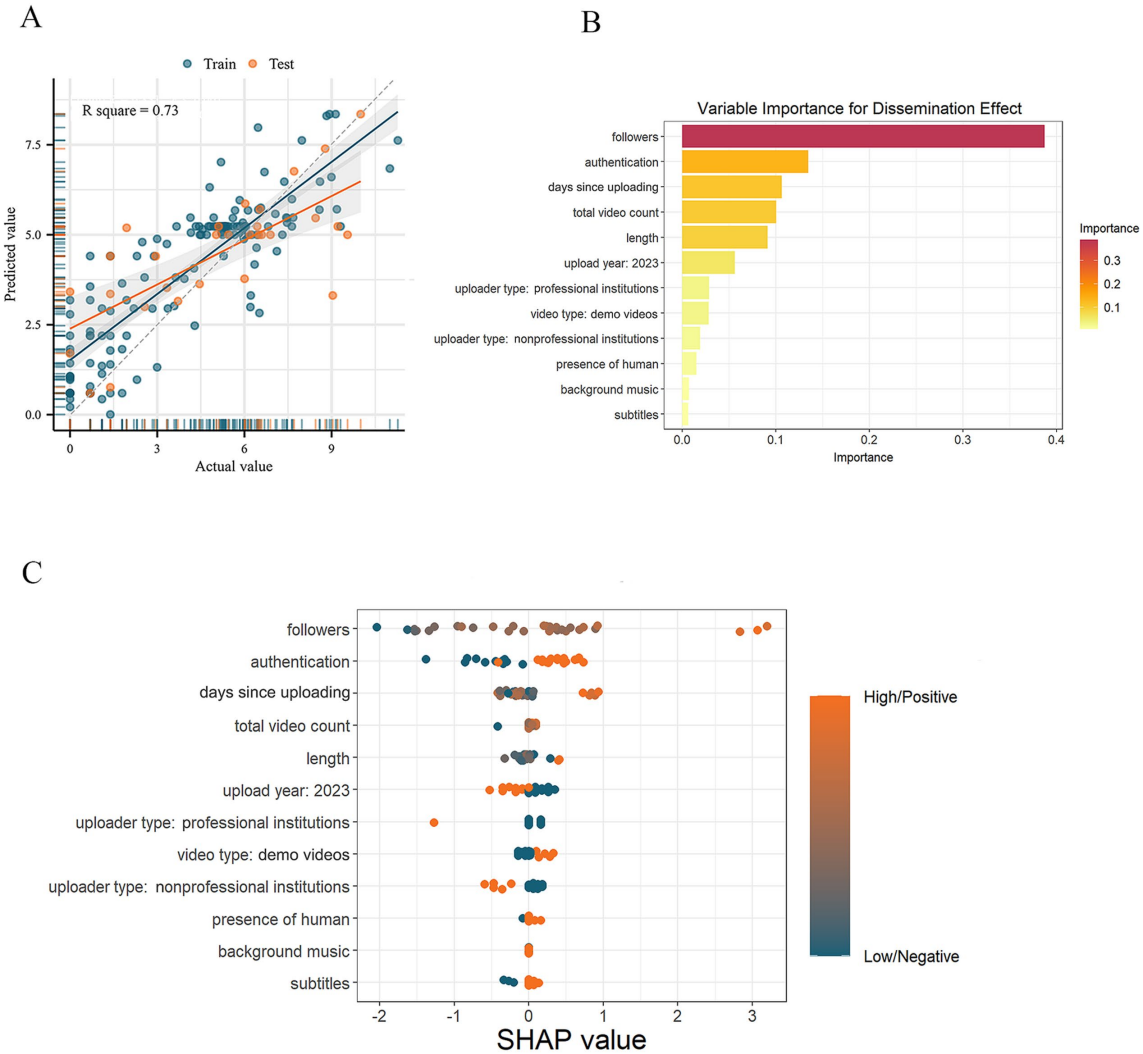
Results of the XGBoost model interpreting the dissemination effect of brain tumor-related short videos. (A) The goodness of fit of the XGBoost model; (B) the importance of variables on the number of likes of short videos; (C) the SHAP value of important variables. XGBoost, the eXtreme gradient boosting; SHAP, SHapley Additive exPlanation.

The following analysis was conducted using data from both platforms to diminish potential bias from the heterogeneity between video sources. Initially, the videos were randomly divided into a training set and a testing set with a ratio of 7:3. The training set was employed to develop the XGBoost model, while the testing set was used to evaluate its predictive performance. The constructed model achieved an R square of 0.73, demonstrating strong predictive performance, as shown in Figure 4A. Figure 4B highlighted the top 12 crucial variables, while the SHAP values offered insights into the impact of these variables on predictions in individual samples, as shown in Figure 4C. Specifically, the number of followers an uploader has was the most influential positive variable in predicting the number of likes of short videos, followed by the authentication status of uploaders. The number of days since uploading and videos uploaded in 2023 had a negative impact on the model, as more recent videos naturally have fewer days since upload. As for the classification of videos, videos uploaded by institutions had a negative association with the number of likes, whereas demo videos were more appealing to the audience. The presence of people, BGM, and subtitles also contributed to the dissemination effect, although their impact was less important. Interestingly, video quality and reliability had only a minor impact on the number of likes.

## 4 Discussion

### 4.1 Principal findings

This is the first cross-sectional study investigating the dissemination effect, quality, and reliability of brain tumor-related short videos on TikTok and Bilibili. The search for “brain tumor” on TikTok hit fewer unrelated short videos than Bilibili. Significant differences were found in almost all characteristics of representative short videos between the two platforms, indicating the heterogeneity between these online data sources. The GQS score and DISCREN reliability instrument were used to assess the quality and reliability of these short videos, which were further classified for subgroup analysis. The median quality and reliability of collected short videos were below average, although these features had little influence on the dissemination. Videos uploaded by professionals and those focused on disease knowledge or case reports exhibited higher video quality and reliability.

### 4.2 Video classification, quality, and reliability

Most importantly, both GQS and DISCERN scores were unsatisfactory. In the GQS assessment, a few short brain tumor-related videos conveyed appropriate and sufficient information about their topics. Their flow was low on Bilibili, which partially contributed to their lower scores than TikTok. Regarding specific questions in the DISCERN reliability instrument, the source of publication (question 4) was usually missing, not to mention the additional supporting information (question 7). The uploading time of videos was regarded as the publication date, while no information on the time of production and revision was provided, which could be important for video clips and demos, as they were likely to be unoriginal publications (question 5). The disclosure of potential bias (question 6) and description of uncertainty (question 8) were usually conducted by statements in the video, such as “for reference only” and “please seek medical attention for any discomfort,” which functioned more as disclaimers than actual disclosures of bias or uncertainty. As for questions 1 (setting aims), 2 (achieving aims), and 3 (relevance), some might be limited by the form of a short video, and some were not intended to disseminate medical knowledge of brain tumors.

Among the video types, short videos about relevant clinical knowledge of brain tumors had the highest scores in both GQS and DISCERN reliability instruments. This is because they usually contained a clear aim and provided appropriate and sufficient information. Demo videos were usually animated introductions of specific treatment technologies, such as craniotomy or stereotactic radiotherapy. These videos only provided general visual information and lacked discussions of indication, contraindication, and their application in different types of brain tumors, resulting in lower scores than videos on disease knowledge and case reports. Case reports were typically clips of outpatient services and were not intended to deliver knowledge purposely. Although some managed to disclose bias and discuss uncertain topics, they inevitably fell short in setting and achieving clear aims. Research and educational videos introduced advancements or practices in certain fields of brain tumor treatment. Although these videos contained medical content and were freely accessible, the information within them was considered less useful for the public and even for professionals in some cases, resulting in lower quality and reliability scores. News reports, documentaries, and other types of videos were uploaded by nonprofessionals and were not for medical purposes. Even when some news reports included interviews with doctors, these videos still received some of the lowest scores.

Videos by professional uploaders had higher quality and reliability, which was consistent with previous studies (29, 32, 34, 35, 39). Regarding diverse types of professional individuals, no significant difference was found in the video quality and reliability between modern and traditional medicine practitioners. From the perspective of modern medicine, most brain tumors, such as intracranial mass lesions, were first treated by craniotomy and tumor resection or biopsy (56). Most of the traditional medicine practitioners in our study were traditional Chinese medicine (TCM) practitioners. Nowadays, the definition of TCM remains ambiguous, and TCM practitioners might also have received education in modern medical courses.

Additionally, the appeal of TCM to the public in China might encourage some uploaders to label themselves as TCM practitioners. Another study found that the quality of short videos uploaded by traditional Chinese medicine professionals was poor (29). More studies on this matter are needed.

Lower quality and reliability were found in short videos on Bilibili compared to those on TikTok. This could be partially explained by the much higher ratio of professional uploaders on TikTok. As medical practitioners, their professional education allowed them to produce video content with higher clinical value and a more prudent perspective. The results from the subgroup analysis of different uploaders also supported the theory. Additionally, it might be relevant that TikTok contained more short videos of clinical knowledge and case reports, which, as previously mentioned, tended to score higher.

### 4.3 Video dissemination

Overall, videos from TikTok excelled in the dissemination effect compared to those from Bilibili due to the nature of short videos, which are fresh, fragmented, and easier to access and interact with. This was also consistent with the larger number of followers that TikTok uploaders had, indicating the greater appeal of short videos in contrast to traditional videos. However, the statistics representing video dissemination varied tremendously even within the same platform, suggesting the diversity of brain tumor-related videos. Demo videos and clips of news and documentaries received more likes, comments, shares, and saves than videos on disease knowledge and case reports on both platforms. The mismatch between the depth of knowledge in short videos and their dissemination effect indicated the need for studies with better design. One possible explanation is that most social media users are healthy individuals who take short videos as entertainment rather than as a source of medical advice. In the prediction model for the dissemination effect of brain tumor-related short videos, the top six variables were all fundamental features of short videos, which accounted for most of the prediction weight. A higher number of followers and uploaded videos of uploaders and a longer time since uploading positively contributed to video dissemination. Authentication status positively contributed to prediction results, possibly due to the distribution differences of these statistics across the two platforms. TikTok had a significantly higher ratio of certified uploaders, and this statistic could easily be influenced by the platform’s specific rules. The presence of people and the addition of BGM and subtitles contributed slightly to the dissemination of short videos. A previous study has found that the presence of people was associated with the number of likes according to a multiple linear regression analysis, while BGM and subtitles were not significant (35). In contrast, quality and reliability were given much less weight in the XGBoost algorithm, which was consistent with previous studies (29, 34, 35).

### 4.4 Different roles in medical-themed short videos

Advances in social media have made it possible for ordinary people to express their views, with short videos emerging as a popular medium. Medical-themed short videos play a significant role due to the widespread demand for medical care. Various participants play different roles in these videos. For patients, the primary purpose is to provide useful, accurate information. However, the majority of short videos related to brain tumors currently lack adequate quality and reliability. Therefore, patients should exercise caution when consuming this content and avoid making medical decisions based on a single source.

For the general audience, motivations for watching these videos vary, ranging from entertainment and accidental discovery to algorithm-driven recommendations. Only a small portion of viewers actively seek medical information, specifically related to brain tumors. Their need for medical-themed short videos should also be considered.

For uploaders, social media serves as an effective tool for enhancing their influence in the field. By uploading massive medical-themed short videos that cater to the audience, uploaders can easily gather followers and gain popularity. The quality and reliability of medical information are not the priority, as uploaders only need to meet the most basic requirements of the platform. This works similarly for medical practitioners, as online influence can also bring positive feedback (57). Although medical professionals can produce high-quality videos, most are not sufficiently motivated, as high quality does not necessarily translate to high influence, as our study and others have shown. Besides, the limited length of short videos makes it difficult to comprehensively cover a topic, as video length is often associated with quality (32).

Furthermore, short videos could be uploaded part-time or primarily produced by professional video producers instead of medical practitioners. Both circumstances might affect the quality and reliability of these videos. The burnout of physicians could affect the quality of patient care, which might also influence the quality and reliability of their video content (58).

For the platform, medical-themed short videos are only one sub-section. However, due to their special role in public health, platforms must regulate them appropriately. First, the uploaders should be categorized as non-professionals who are unqualified to provide medical services. Second, restrictions on commercial advertising in these videos must be enforced to reduce biased information (59). Third, creating an official web page or account providing peer-reviewed medical information is encouraged, and it should be prioritized in search results when medical terms are queried. For authorities, social media plays an important role in public health and may also be helpful in cancer screening and early diagnosis (60). The National Natural Science Foundation of China has emphasized the importance of scientific popularization (61). The popularization of medical knowledge through short videos is one part of this effort. Additionally, laws and regulations against misinformation on the Internet could improve the overall quality of online medical information. The supervision of medical information on short video platforms should be strengthened.

Furthermore, health campaigns targeting specific populations could be launched (62). For researchers, one important task is establishing widely acknowledged medical information standards in short videos. The most commonly used tools, including DISCERN (8), GQS (9), and JAMA criteria (7), were established long before the prevalence of short videos. In summary, the quality and reliability of medical-themed short videos result from joint efforts. More studies are needed to improve the quality and reliability of medical-themed short videos while enhancing their dissemination effect.

### 4.5 Limitations

First, the scope of our study is confined to two popular short-form video platforms, which may limit the generalizability of our findings to other platforms or social media environments. Second, the cross-sectional design of our study restricts our ability to infer causal relationships between the identified factors and video dissemination. Additionally, our results have regional specificity, as we did not analyze brain tumor-related short videos beyond the Chinese Internet. Moreover, our use of the XGBoost model, while powerful in handling complex data, has its challenges. The model’s predictions are inherently dependent on the quality of the input data, and any biases or inaccuracies in the data can affect the reliability of the results. Despite employing SHAP for interpretability, the complexity of the model may still pose challenges for those less familiar with machine learning techniques.

These limitations underscore the need for further studies that explore additional platforms, employ longitudinal designs, and investigate alternative machine-learning approaches to validate and extend our findings. Future research should also consider incorporating more diverse data sources to capture a broader range of brain tumor-related content and its dissemination dynamics across various social media platforms.

## 5 Conclusion

This study evaluated the quality, reliability, and dissemination of brain tumor-related short videos on TikTok and Bilibili, highlighting significant shortcomings. Our analysis showed that while videos from professional sources, particularly those focused on disease knowledge, scored higher in quality and reliability, the number of followers an uploader had was the primary factor driving a video’s dissemination. This suggests that content quality alone has a limited impact on viewer engagement. However, our analysis was limited to two platforms featuring Chinese videos. Future research should broaden the scope to better understand the factors influencing the dissemination factors of medical-themed videos.

## Data Availability

The raw data supporting the conclusions of this article will be made available by the authors without undue reservation.
